# Research progress on the mechanisms underlying poultry immune regulation by plant polysaccharides

**DOI:** 10.3389/fvets.2023.1175848

**Published:** 2023-04-17

**Authors:** Ruo-Han Zhao, Fang-Xiao Yang, Yi-Cheng Bai, Jing-Ying Zhao, Mei Hu, Xin-Yan Zhang, Teng-Fei Dou, Jun-Jing Jia

**Affiliations:** ^1^College of Animal Science and Technology, Yunnan Agricultural University, Kunming, Yunnan, China; ^2^College of Animal Science and Veterinary Medicine, Yunnan Vocational and Technical College of Agriculture, Kunming, Yunnan, China; ^3^Kunming CHIA TAI Co., Ltd., Kunming, Yunnan, China

**Keywords:** plant polysaccharides, immune regulation, intestinal health, immune adjuvant, poultry

## Abstract

With the rapid development of poultry industry and the highly intensive production management, there are an increasing number of stress factors in poultry production. Excessive stress will affect their growth and development, immune function, and induce immunosuppression, susceptibility to a variety of diseases, and even death. In recent years, increasing interest has focused on natural components extracted from plants, among which plant polysaccharides have been highlighted because of their various biological activities. Plant polysaccharides are natural immunomodulators that can promote the growth of immune organs, activate immune cells and the complement system, and release cytokines. As a green feed additive, plant polysaccharides can not only relieve stress and enhance the immunity and disease resistance of poultry, but also regulate the balance of intestinal microorganisms and effectively alleviate all kinds of stress faced by poultry. This paper reviews the immunomodulatory effects and molecular mechanisms of different plant polysaccharides (*Atractylodes macrocephala* Koidz polysaccharide, *Astragalus* polysaccharides, Taishan *Pinus massoniana* pollen polysaccharide, and alfalfa polysaccharide) in poultry. Current research results reveal that plant polysaccharides have potential uses as therapeutic agents for poultry immune abnormalities and related diseases.

## 1. Introduction

The poultry industry is perennially plagued by many diseases, including infectious bursal disease, *Salmonella*, avian influenza, Newcastle disease, black lung, and mixed infections of various diseases, with a new disease observed in China's poultry industry almost every two years (1–4). In addition, poultry farming faces a considerable number of environmental stresses daily, including stressors, toxic substances, drug effects, inadequate nutritional factors, viruses, and diseases, which lead to low immune levels or dysplastic immune organs, resulting in abnormal immune function (5–8). Owing to imperfections in the immune system, the immune barrier of the body is easily broken and certain pathogenic factors can easily invade the body (9, 10). The widespread prevalence of many epidemic diseases, limited knowledge on their pathogenesis, and high feeding density of rural chicken farms have led to the abuse of antibiotics, which has led to unhealthy practices in the poultry industry and huge economic losses (11–13).

With prohibitions on antibiotics use and the promotion of green farming, replacing antibiotics with green additives has become a research hotspot at home and abroad. Many studies have shown that plant polysaccharides can regulate the growth and development of immune organs and enhance the body's resistance to bacteria and viruses (14, 15). Polysaccharides are key factors in cell surface signal recognition, cell-to-cell signal transmission, and immune-related responses, and they can stimulate innate and cellular immunity through interactions with immune cells, regulate the immune response in an appropriate manner, and promote the immune system against a variety of adverse factors (16).

The immune organs in poultry are mainly composed of the thymus, spleen, and bursa of Fabricius. Immune organs are sites where various immune cells differentiate, mature, and produce an immune response. These immune cells include macrophages, dendritic cells, T lymphocytes, and B lymphocytes (17, 18). Immune cells produce a variety of cytokines and chemokines that enhance the immune response and protect the body (19). In addition, improving immunity is an effective strategy to reduce disease susceptibility and enhance defense against pathogens, and stimulating macrophages, lymphocytes, dendritic cells, and other immune cells plays a vital role in enhancing immunity (20, 21). Many previous studies have shown that plant polysaccharides can regulate immunity by increasing the index of immune organs and activating cellular and humoral immunity (22). In addition, plant polysaccharides can alleviate oxidative stress, liver injury, and immunosuppression induced by various adverse conditions. This study mainly reviewed the effect of plant polysaccharides on poultry immune regulation and the underlying molecular mechanisms to provide a reference basis for the application of these substances in poultry breeding.

## 2. Plant polysaccharide

### 2.1. *Atractylodes macrocephala* koidz polysaccharide

**Figure 1:**
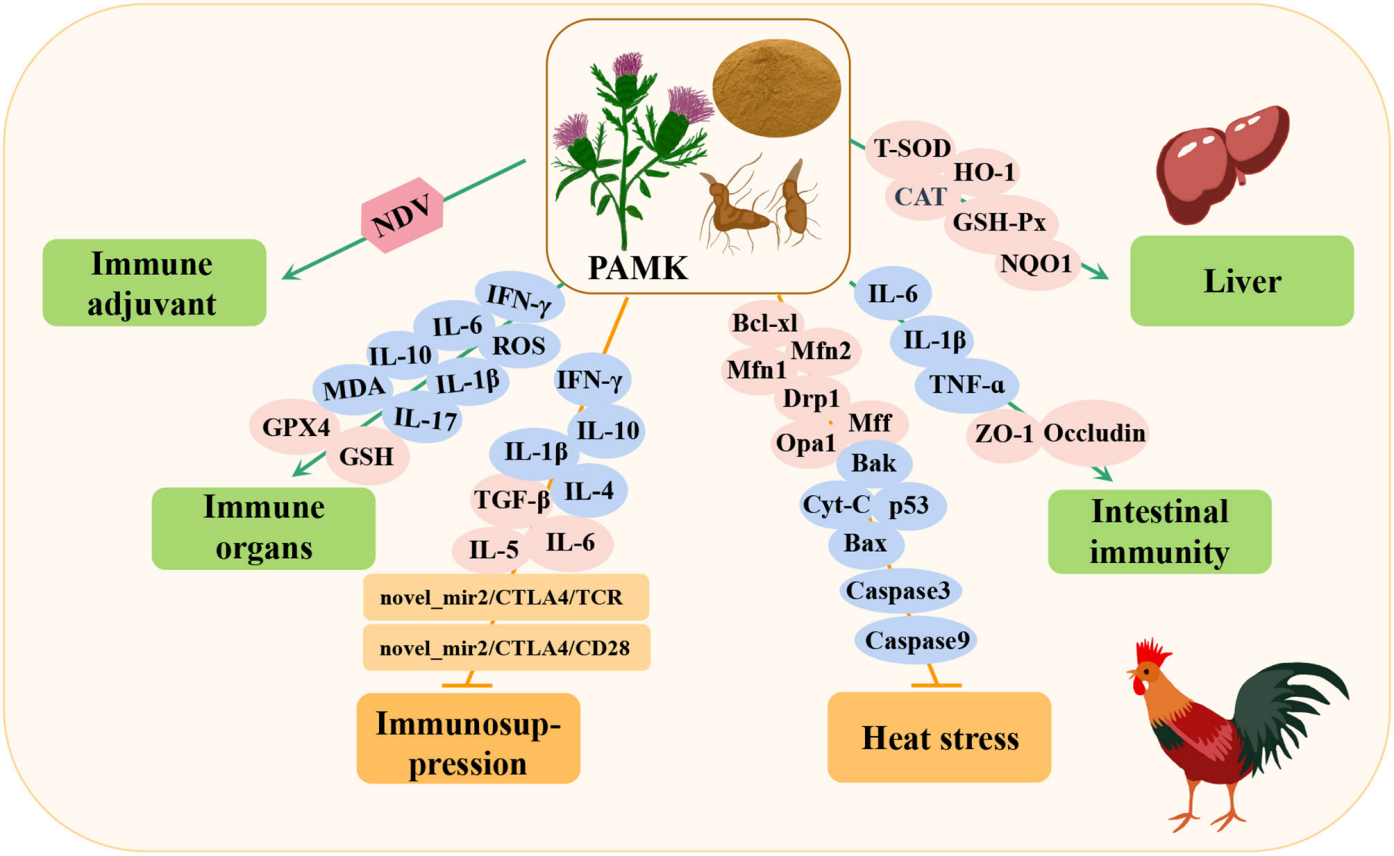
Immunomodulation and protection of immune organs by *Atractylodes macrocephala* Koidz polysaccharide (PAMK) in poultry. NDV, newcastle disease virus; IFN-γ, interferon-gamma; IL-6, interleukin-6; IL-10, interleukin-10; MDA, malonaldehyde; ROS, reactive oxygen species; IL-1β, interleukin 1 beta; IL-17, interleukin-17; GSH, glutathione; GPX4, glutathione peroxidase 4; IL-4, interleukin-4; TGF-β, transforming growth factor-β; IL-5, interleukin-5; Bcl-xl, BCL2-like 1; Mfn1, mitofusin 1; Mfn2, mitofusin 2; Drp1, dynamin-related protein 1; Opa1, mitochondrial dynamin like GTPase; Mff, mitochondrial fission factor; Bak, BCL2 antagonist/killer 1; Bax, BCL2-associated x; Cyt-C, cytoplasmic cytochrome c; p53, tumor protein p53; TNF-α, tumor necrosis factor-α; ZO-1, zonula occludes protein; CAT, catalase; HO-1, heme oxygenase 1; GSH-Px, glutathione peroxidase; NQO1, NAD(P)H quinone dehydrogenase 1; T-SOD, total superoxide dismutase.

The polysaccharide of the traditional Chinese medicine *Atractylodes macrocephala* Koidz (PAMK) is an important component of this plant. The main extraction methods are hot water extraction, alcohol precipitation, and ultrasonicassisted enzymatic extraction (23, 24). Different extraction methods produce different components, including arabinose, glucose, galactose, and mannose (25, 26). PAMK is considered a potential therapeutic drug for various diseases in livestock industry because of its biological activities, such as the promotion of immunomodulation (27), anti-tumor (28), anti-inflammation (29), and hypoglycemic effects (30) (Table 1). Importantly, recent studies have found that PAMK plays a useful role in poultry breeding, including alleviating immunosuppression, reducing heat stress damage, maintaining immune organs, liver functions, and intestinal barriers, and improving vaccine efficacy (Figure 1).

**Table 1:** Structure and structure-activity-relationship of polysaccharide from *Atractylodes macrocephala* Koidz.

**Type**	**Extraction method**	**Monosaccharide composition**	**Molecular weight/kDa**	**Structure characteristics**	**Function**	**Reference**
*Atractylodes macrocephala* Koidz	Ultrasonicassisted enzymatic extraction	-	-	-	Anti-oxidation	(23)
*Atractylodes macrocephala* Koidz	Alcohol precipitation	Gal: Ara: Glc=1.00: 1.50: 5.00	4.10 kDa	Three α-pyranose residues, α D Glu*p*α D Gal*p* and α L Ara*f*	Anti-tumor	(25)
*Atractylodes macrocephala* Koidz	Hot water extraction	Man: GalA: Glc: Gal: Ara=1.00: 8.58: 27.28: 3.68: 4.99	4.35 kDa	→ 3-β-D-Glc*p*-(1 → , → 3,6-β-D-Glc*p*-(1 → , → 6-β-D-Glc*p*-(1 → ,T-β-D-Glc*p*, → 4-α-Gal*p*A-(1 → , → 4-α-Gal*p*A-OMe-(1 → , → 5-α-Ara*f*-(1 → , → 4,6-β-man*p*-(1 → and → 4-β-Gal*p*-(1 →	Immunomodulation	(26)
*Atractylodes macrocephala* Koidz	Hot water extraction	Rha: Ara: Gal: GalA=1.00: 1.84: 0.74: 4.23	161.90 kDa	t-Ara*f*, 1,5-Ara*f*, 1,3,5-Ara*f*, 1,2-Rha*p*, 1,2,4-Rha*p*, t-Gal*p*, 1,4-Gal*p*, 1,3,4-Gal*p*, 1,4,6-Gal*p* and 1,4-Gal*p*A residues	Immunomodulation	(27)
*Atractylodes macrocephala* Koidz	Alcohol precipitation	Ara: Glc=1.00: 4.57	2.10 kDa	Pyranose rings and α-type and β-type glycosidic lin kages	Anti-tumor	(28)
*Atractylodes macrocephala* Koidz	Hot water extraction	Fuc: Ara: Gal: Glc: Xyl: Man: Fru: GalA=0.07: 0.99: 1.23: 6.70: 0.01: 0.09: 0.12: 0.76	4.75 kDa	-	Anti-inflammation	(29)
*Atractylodes macrocephala* Koidz	Water extraction	Glc: Gal: Man: Ara: Rha=3.00: 2.50: 1.30: 3.50: 1.00	-	-	Hypoglycemic	(30)
*Atractylodes macrocephala* Koidz	Alcohol precipitation	Glc: Man=0.58: 0.42	2.82 kDa	(1 → 3)-Glc residues. (1 → 6)-linked monosaccharide backbone	Hypolipidemic	(31)

Gal, galactose; Ara, arabinose; Glc, glucose; Man, mannose; GalA, galacturonic acid; Rha, rhamnose; Fuc, fucose; Xyl, xylose; Fru, fructose; GlcA, glucuronic acid; GlcN, glucosamine; GalN, galactosamine; Rib, ribose; Myo, myo-inositol; ManA, mannuronic acid.

Studies have shown that immunosuppression in poultry is very common and usually caused by infection, stress, and abuse of antibiotics and chemicals. In the poultry immunosuppression model, PAMK can reduce damage to immune organs, alleviate the decreased function and imbalances of immune cells, maintain the balance of immune cytokines, and help restore the morphology of immune organs (32–34). In addition, heat stress can lead to immune organ dysfunction, apoptosis, and oxidative damage in poultry, resulting in decreased immunity. However, PAMK can improve immune system dysfunction and oxidative damage caused by heat stress by increasing the level of cytokines, reducing oxidative stress, enhancing mitochondrial function, and inhibiting apoptosis (35–37). Studies have shown that PAMK can alleviate the injury to immune organs and enteritis-associated inflammation induced by lipopolysaccharides (LPSs), reduce the level of inflammatory factors, and improve disorders among intestinal flora (31, 38). In addition, the poultry liver is the largest reticular endothelial cell phagocytosis system, and it plays an irreplaceable role in immune function through phagocytosis, isolation, and elimination of invasive and endogenous antigens. However, PAMK can improve liver injury by regulating the activity of antioxidant enzymes and liver lipid metabolism (39). Studies have also shown that PAMK can increase the antibody titer of poultry vaccinated with the Newcastle disease (ND) vaccine (40, 41).

At the molecular level, PAMK can activate T lymphocytes in the thymus through the novel_mir2/CTLA4/TCR and novel_mir2/CTLA4/CD28 signaling pathways and inhibit the transcription of proinflammatory cytokines, such as interleukin 1 beta (IL-1β), interferon-gamma (IFN-γ), interleukin-4 (IL-4), and interleukin-10 (IL-10). Increased expression of cytokines, such as transforming growth factor-β (TGF-β), interleukin-6 (IL-6), and interleukin-5 (IL-5), maintains the balance of cytokines in the body to alleviate immunosuppression (32–34). In a heat stress model, PAMK was shown to maintain the number of mitochondria and reduce apoptosis by increasing the expression of the Mff, Drp1, Opa1, Mfn1, and Mfn2 genes (35). Moreover, the expression of p53, Cyt-C, Bax, Bak, Caspase3, and Caspase9 proapoptotic genes increased the expression of anti-apoptotic Bcl-xL gene and alleviated the expression of heat shock proteins, such as heat shock protein 60 (HSP60) and HSP70, to prevent further injury caused by heat stress, relieve the state of heat stress, and improve immunity (36, 37). In addition, PAMK scavenges lipid peroxidation reactive oxygen species (ROS) and malonaldehyde (MDA) by increasing the expression of GPX4, increasing the content of its cofactor glutathione (GSH), and reducing the expression of proinflammatory cytokines IFN-γ, IL-1β, IL-6, IL-10, and IL-17, thereby improving the antioxidant capacity of the body and reducing immune organ damage caused by LPSs (31). Similarly, PAMK increases tight junction occlusal proteins and ZO-1, reduces the levels of endotoxins, CRP, and proinflammatory factors (IL-1β, TNF-α, and IL-6), and improves the stability of intestinal flora abundance, thereby alleviating intestinal damage and microbial disorders caused by LPSs (38). In addition, Miao et al. demonstrated that PAMK can reduce liver injury by reducing oxidative stress-related insulin resistance or by directly changing the expression of genes involved in liver lipid metabolism, such as catalase (CAT), heme oxygenase 1 (HO-1), glutathione peroxidase (GSH-Px), and NQO1 (39). These studies demonstrated that PAMK plays an important role in immune regulation in poultry and mediates protection against immune organ injury.

### 2.2. *Astragalus* polysaccharide

**Figure 2:**
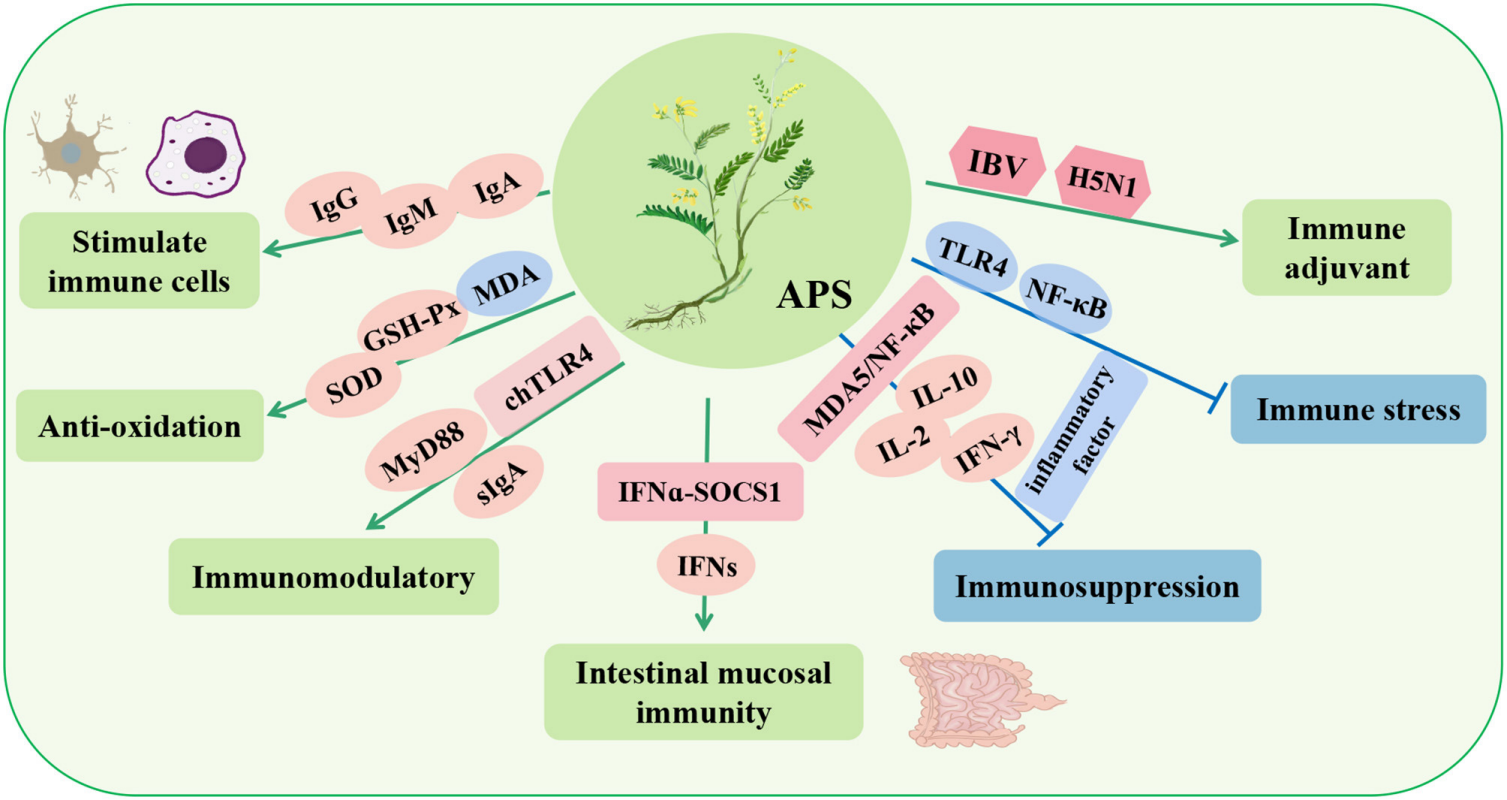
Immunomodulatory effects and mechanisms of *Astragalus* polysaccharide (APS) in poultry. IgA, immunoglobulin A; IgM, immunoglobulin M; IgG, immunoglobulin G; SOD, superoxide dismutase; MyD88, myeloiddifferentiationfactor88; chTLR4, toll-like receptor 4; sIgA, secretory immunoglobulin A; IFNs, type-I-interferon; NF-κB, nuclear factor-κB; MDA5, interferon induced with helicase C domain 1; IL-2, interleukin-2; IBV, infectious bronchitis virus; H5N1, avian influenza.

*Astragalus* polysaccharide (APS) is one of the most important natural active components in *Astragalus*, and it is a water-soluble heteropolysaccharide extracted from the stem or dry root of *Astragalus* and one of the most important natural active components of this plant. APS extraction methods mainly include hot water extraction, cold water extraction, alcohol precipitation and enzyme-assisted extraction (42, 43). The components of different extraction methods are also slightly different, including mannose, glucose, xylose, arabinose, galactose, glucuronic acid, and rhamnose (42, 44). APS has many beneficial properties, such as anti-tumor (44), antioxidant (45), immunomodulatory (46, 47), anti-inflammatory (48), hypoglycemic (49), and other biological activities (Table 2). At present, the immunomodulatory function of APS has received increasing research attention, especially in poultry breeding (Figure 2).

**Table 2:** Structure and structure-activity-relationship of polysaccharide from *Astragalus*.

**Type**	**Extraction method**	**Monosaccharide composition**	**Molecular weight/kDa**	**Structure characteristics**	**Function**	**References**
*Astragalus*	Hot water extraction	All consisted of Ara	40.10 kDa	-	Immunomodulation	(42)
*Astragalus*	Enzyme-assisted extraction	-	-	-	Anti-oxidation	(43)
*Astragalus*	Cold water extraction	Fuc: Ara: Gal: Glc: Xyl=0.01: 0.06: 0.20: 1.00: 0.06	12.30 kDa	α-D-pyranoid configuration	Anti-tumor and immunomodulation	(44)
*Astragalus*	-	Glc: Ara: Rha: Gal=13.90: 8.20: 1.20: 1.00	4.72 kDa	(1 → 4)-D-glucose as the main chain and (1 → 6)-D-glucose linkage as the branch chain	Anti-oxidation	(45)
*Astragalus*	Hot water extraction	Man: Glc: Xyl: Ara: GlcA: Rha=0.27: 12.83: 1.63: 0.71: 1.04: 0.56	2047.76 kDa	Acidic polysaccharides mainly composed of glucose	Immunomodulation and anti-inflammatory	(46)
*Astragalus*	Hot water extraction	Rha: GalA: Gal: Glc: Ara=0.14: 0.14: 9.60: 24.04: 1.00	10.00 kDa	A main chain of−2,3)-α-L-Rha-(1 → , → 5)-α-L-Ara-(1 → , → 3,4)-β-D-Gal-(1 → , → 6)-β-D-Gal-(1 → , → 4)-α-D-Glu-(1 → , → 3,4,6)-β-D-Glu-(1 →	Immunomodulation	(47)
*Astragalus*	Alcohol precipitation	Rha: GalA: Gal: Ara: Glc: GlcA: Fuc=14.47: 49.55: 11.87: 21.47: 0.79: 1.16: 0.44	3373.20 kDa	1,2,3-β-Rha*p*, 1,4-β-Gal*p*A, 1,2,6-β-Gal*p*, 1,4-β-Gal*p*, 1,2-α-Gal*p*A, 1,2-β-Gal*p*, 1,3,5-α-Ara*f*, 1,4-β-Gal*p*A, T-α-Ara*f*, T-β-Rha*p*	Anti-inflammatory	(48)

Studies have shown that APS can stimulate the proliferation of T and B lymphocytes, promote the maturation of macrophages (50) and dendritic cells (51), and exert immune functions. Similarly, dietary APS significantly increases the activities of superoxide dismutase (SOD) and GSH-Px; increases the levels of serum immunoglobulin G (IgG), immunoglobulin M (IgM), and immunoglobulin A (IgA); and decreases the content of MDA, thus improving the immune response of broilers (52). Zhang et al. (53) found that APS upregulated chTLR4 in the bursa, MyD88-independent genes in downstream molecules, and secretory immunoglobulin A (sIgA) in the gut, thus demonstrating that APS can play an immunoregulatory role by activating the chTLR4 pathway (53). Interestingly, continuous inclusion in the male parent diet of APS (10 g/kg/d) could upregulate the serum level of type-I-interferon (IFNs) in the offspring, and APS could induce the intergenerational endotoxin tolerance-like effect and enhance the intestinal mucosal immunity of the offspring by activating the IFNα-SOCS1 pathway (54, 55).

In addition, in a poultry immunosuppression model induced by the heavy metal cadmium (Cd), APS effectively alleviated the immune imbalance induced by Cd by regulating the MDA5/NF-κB signaling pathway, reducing the expression of proinflammatory cytokines (IL-1β, IL-6, and TNF-α), and increasing the activities of antioxidant enzymes (SOD and GSH-Px) (56). Similarly, APS can stimulate the proliferation of lymphocytes and goblet cells of the intestinal epithelium in poultry, upregulate the mRNA expression of IL-2, IL-10, and IFN-γ, and increase the height of villi and the content of lgA, thus alleviating the immunosuppression of the intestine and body (57, 58). Liu et al. (59) also found that APS can reduce the transcription of TLR4 and NF-κB to inhibit the expression of proinflammatory cytokines, thereby improving the immune stress response (59). In addition, APS can be used as a vaccine adjuvant for avian infectious bronchitis virus (IBV**)** and H5N1 avian influenza. It can increase the titer of specific antibodies and the number of lymphocytes and increase the mRNA expression levels of IL-1β, IL-2, IL-8, and TNF-α in a dose-dependent manner to better prevent virus infection (60, 61). These studies showed that APS can enhance immune regulation, improve immunosuppression, and enhance vaccine titers in poultry.

### 2.3. Taishan *Pinus massoniana* pollen polysaccharide

**Figure 3:**
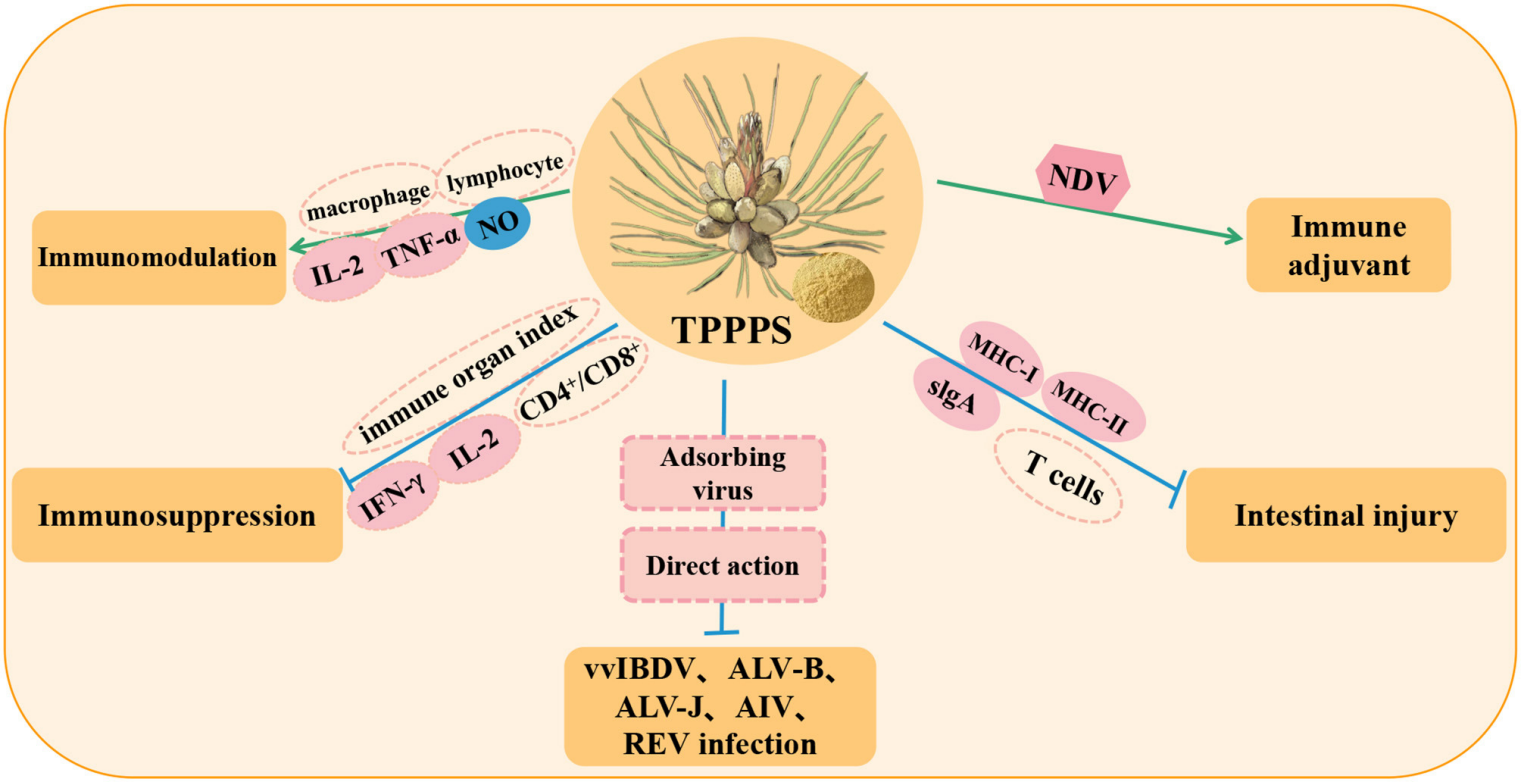
Immunomodulation and antiviral effects of Taishan *Pinus massoniana* pollen polysaccharide (TPPPS) in poultry. NO, nitrous oxide; vvIBDV, very virulent infectious bursal disease virus; ALV-B, avian leukosis virus subgroup B; ALV-J, subgroup J avian leucosis virus; AIV, avian influenza virus; REV, reticuloendotheliosis virus; MHC-I, major histocompatibility complex class I; MHC-II, major histocompatibility complex class II.

Taishan *Pinus massoniana* pollen polysaccharide (TPPPS) is obtained from Taishan *Pinus massoniana* pollen *via* water extraction and ethanol precipitation, and it has multiple effects. It is composed of mannose, ribose, xylose, glucuronic acid, galacturonic acid, glucose, galactose, and arabinose (62). Many studies have shown that TPPPS has a wide range of biological activities, such as antioxidation, immune regulation, antivirus, and liver protection activities (63, 64) (Table 3). In addition, an increasing number of studies has found that TPPPS has a variety of beneficial effects on immune regulation in poultry (Figure 3).

**Table 3:** Structure and structure-activity-relationship of polysaccharide from Taishan *Pinus massoniana* pollen.

**Type**	**Extraction method**	**Monosaccharide composition**	**Molecular weight/kDa**	**Structure characteristics**	**Function**	**References**
Taishan *Pinus massoniana* pollen	Hot water extraction and ethanol precipitation	Man: Rib: GluA: GalA: Glc: Gal: Ara=1.35: 2.15: 1.42: 0.38: 2.01: 1.46: 1.23	56.00 kDa	-	Anti-oxidation	(62)
Taishan *Pinus massoniana* pollen	Hot water extraction and ethanol precipitation	Man: Rib: Xyl: GluA: GalA: Glc: Ara=0.29: 1.35: 1.85: 1.52: 1.03: 1.33: 2.63	128.00 kDa	-	Immunomodulation and antiviral	(62)
Taishan *Pinus massoniana* pollen	Hot water extraction and ethanol precipitation	Man: Rha: GlcA: GalA: Glc: Gal: Xyl: Ara=0.18: 0.17: 0.08: 0.54: 6.92: 0.52: 0.56: 1.04	-	94.7% total carbohydrate	Anti-oxidation and hepatoprotective	(63)

A study reported that TPPPS can promote the proliferation and activation of poultry macrophages and enhance poultry resistance to pathogens by stimulating and activating lymphocytes and immunomodulatory functions by releasing cytokines IL-2 and TNF-α and increasing the chemical signaling mediator NO (65). Simultaneously, a proteomic study on peripheral blood lymphocytes of chickens treated with TPPPS showed that the differentially expressed proteins in the TPPPS group were related to host innate immune response and stress-induced immune response (66). The study also showed that in the model of poultry immunosuppression, TPPPS significantly enhanced the immune organ index of chickens, which enhanced the immune ability, promoted the differentiation and proliferation of CD4^+^ and CD8^+^T lymphocytes, and significantly increased the levels of cytokines IL-2 and IFN-γ to mediate the immune response and enhance the immune system function of chickens (67, 68). In addition, in an intestinal injury model, TPPPS promoted the renewal of intestinal epithelial cells and induced intestinal mucosal immunity to produce a large amount of slgA to form a protective barrier and activate the intestinal mucosal immune system. The antigen presentation molecule MHC can present antigens to helper T cells and activate adaptive immunity, while TPPPS promotes the increase of MHC-I genes (BF1 and BF2) and MHC-II genes (BLA, BLB1, and BLB2) in intestinal tissue. Thus, upregulated MHC promotes antigen presentation, drives the activation of immature T cells (naive T), and rapidly differentiates effector T cells, regulatory T cells, cytotoxic T cells, and memory T cells to induce an immune response, thereby alleviating intestinal injury in poultry (69).

In addition, TPPPS can effectively inhibit infection by a variety of poultry viruses and bacteria, including infectious bursal disease virus (vvIBDV) (70), avian leukosis virus subgroup B (ALV-B) (62), subgroup J avian leukosis virus (ALV-J) (71–73), H9N2 subtype avian influenza virus (AIV) (74), reticuloendotheliosis virus (REV) (67), *Proteus mirabilis* (75), and *Bordetella avium* (76). Many studies have shown that TPPPS can effectively inhibit viral infection and host cell replication and significantly reduce virus shedding and viral load in immune organs. The antiviral mechanism may be the binding of TPPPS to the adsorption site of virus particles on the cell surface, the inhibition of virus adsorption to cells by the direct interaction between the virus and polysaccharide, or both effects at the same time. TPPPS can also be used as a vaccine adjuvant to improve the antibody titer of poultry vaccinated with ND vaccine (68). Moreover, TPPPS mixed with pathogen vaccine and inoculated into chicken significantly increased the serum antibody response and immune protection against bacterial infection (75). These results suggest that TPPPS can improve the immune system, promote the immune response, and enhance the activity of lymphocytes, thus indicating that it is a potential immune enhancer against viral and bacterial infections.

### 2.4. Alfalfa polysaccharide

**Figure 4:**
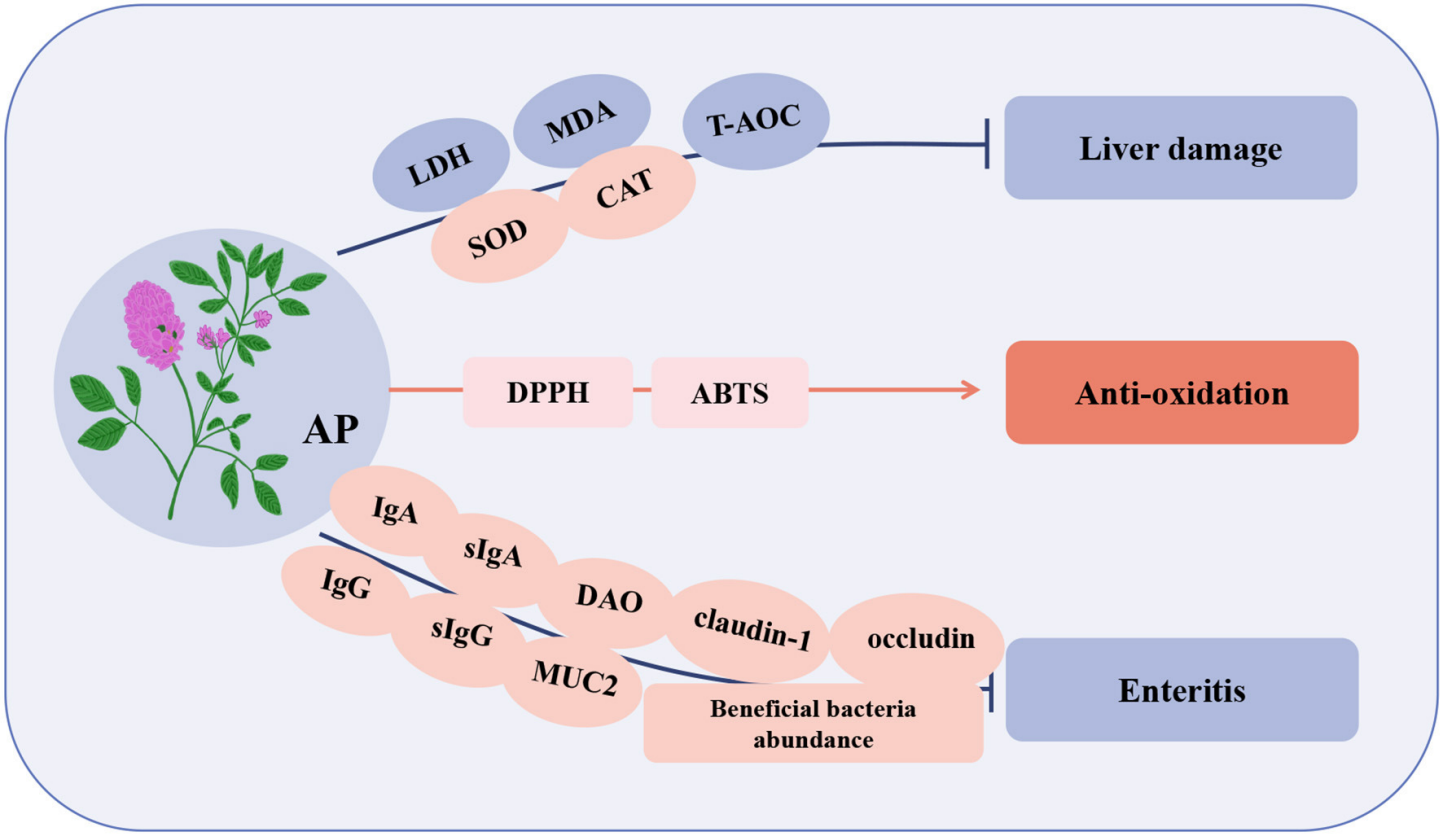
Immunomodulatory effects of Alfalfa polysaccharide (AP) in poultry. LDH, lactate dehydrogenase; T-AOC, total antioxidant capacity; DPPH, 2,2-diphenyl-1-picrylhydrazyl; ABTS, 3-ethylbenzothiazoline-6-sulfonate; sIgG, secretory immunoglobulin G; DAO, diamine oxidase; MUC2, mucin 2.

Alfalfa polysaccharide (AP) is a bioactive compound extracted from alfalfa, and it can be extracted by hot water extraction, alcohol precipitation and complex enzyme-assisted extraction (77–79). The components extracted by each method are slightly different and include caramel, fucose, arabinose, glucose, galactose, glucuronic acid, rhamnose, and mannose. AP has many biological functions, such as immune regulation (77), anti-inflammation (80), antioxidation (79), anticancer (81), and neuroprotective (82) (Table 4). AP has been reported to promote the proliferation of lymphocytes and dendritic cells and enhance the killing activity of NK cells in mice (83). It can also enhance the immune function of macrophages by promoting their proliferation and stimulating the secretion of NO, TNF-α, and IL-6 through the MAPK and NF-κB signaling pathways (84). Similarly, AP can activate B cells through the TLR4-MyD88 signaling pathway and increase B cell IgM production through the MAPK/p38 pathway (85). Recently, many researchers have reported that AP has a significant effect on poultry (Figure 4).

**Table 4:** Structure and structure-activity-relationship of polysaccharide from Alfalfa.

**Type**	**Extraction method**	**Monosaccharide composition**	**Molecular weight/kDa**	**Structure characteristics**	**Function**	**References**
Alfalfa	Water extraction and alcohol precipitation	Fuc: Ara: Gal: Glc: Xyl: Man: GalA: GlcA=2.60: 8.00: 4.70: 21.30: 3.20: 1.00: 74.20: 14.90	3300.00 kDa	1,5-Araf, T-D-Glc, T-D-Gal, 1,4-Gal-Ac, 1,4-Glc, 1,6-Gal, 1,3,4-GalA	Immunomodulation	(77)
Alfalfa	Complex enzyme-assisted extraction	-	-	-	Anti-oxidation	(78)
Alfalfa	Hot water extraction	Rha: Xyl: Ara: GalA: Man: Glc=2.13: 3.07: 2.77: 1.00: 1.30: 1.10	10.00 kDa	→ 2)-α-L-Rha*p*-(1 → , α-D-GalA*p*-(1 → , → 4)-β-D-Man*p*-(1 → , → 5)-α-L-Ara*p*-(1 → , → 3)-β-D-Xyl*p*-(1 → , → 6)-β-D-Glc*p*-(1 →	Anti-oxidation and anti-tumor	(79)
Alfalfa	Hot water extraction	Rha: Xyl: GalA: Man: Glc: Gal=12.50: 16.00: 23.75: 31.75: 46.75: 1.00	15.80 kDa	→ 3)-α-L-Rha*p*-(1 → , → 2)-α-L-Rha*p*-(1 → , → 6)-α-D-Gal*p*-(1 → , α-D-GalA*p*-(1 → , α-D-Glc*p*-(1 → , → 3)-β-D-Man*p*-(1 → , → 3)-β-D-Xyl*p*(1 →	Anti-oxidation and anti-tumor	(79)
Alfalfa	Hot water extraction	Rha: Ara: Man: Gal=0.44: 0.13: 0.11: 0.32	13.40 kDa	2-O-acetyl-β-D-Man*p*-(1 → 3)-α-L-Ara*f*-1(1 → 4)-α-L-Rha*p*-(1 → , → 3)-α-L-Rha*p*-(1 → 4)-α-L-Rha*p*-(1 →	Neuroprotective	(82)
Alfalfa	Hot water extraction	Rha: Ara: Man: Gal=0.50: 0.22: 0.07: 0.21	11.20 kDa	→ 3)-α-L-Rha*p*-(1 → 4)-α-L-Rha*p*-(1 → , → 3)-α-D-Gal*p*-(1 → 4)-α-D-Gal*p*-(1 → , β-D-Man*p*-(1 → 2)-O-acetyl-β-D-Man*p*-(1 → , β-D-Man*p*-(1 → 3)-α-L-Rha*p*-(1 →	Anti-oxidation	(82)

A study reported that AP can significantly promote the proliferation of lymphocytes in blood and spleen of broilers, and the effect of 20 μg/mL is the best. Compared with the control group, the T and B lymphocytes of 42-day-old spleen increased by 38.37 and 19.89%, respectively (86). In poultry breeding, *Salmonella* causes enteritis, decreased immunity, and organ damage in poultry and is also a zoonotic pathogen that is very harmful to the poultry industry (87). After poultry infected with *Salmonella* were fed AP, the contents of IgG and IgA in serum, sIgA and sIgG in duodenal mucosa, the activity of diamine oxidase (DAO) in intestine and the expression of tight junction protein (claudin-1, occludin, and MUC2) in jejunum increased, indicating that AP improved the immune status and intestinal barrier function of broilers. In addition, AP promotes the abundance of intestinal beneficial bacteria (*Bacteroides, Pasteurella, Butyricomonas*, and *Puccinellidae*) increased, the number of core bacteria related to body weight increased, and the intestinal morphology and intestinal mucosal barrier function were improved, thus improving the overall resistance of poultry (88). Studies have shown that the liver is an important organ for detoxification and immune regulation, and its health is a fundamental indicator of poultry health. An oxidative stress model of hepatocytes induced by hydrogen peroxide (H_2_O_2_) *in vitro* showed that lactate dehydrogenase (LDH) and MDA are sensitive indicators of oxidative damage in hepatocytes while AP induces a decrease in LDH and MDA, thus protecting the integrity of the hepatocyte membrane and normal oxidative state. At the same time, AP overcomes the oxidative damage induced by H_2_O_2_ by increasing the enzyme activities of SOD and CAT and the non-enzymatic total antioxidant capacity (T-AOC) of hepatocytes, thus protecting the hepatocytes of laying hens (89). Studies have shown that the antioxidant activity of natural compounds can be reflected by the scavenging of DPPH and ABTS free radicals. However, AP has strong scavenging ability for DPPH and ABTS free radicals, indicating that AP has high antioxidant activity (79). In summary, AP has the advantages of enhancing immune regulation and promoting growth performance and antioxidation, and it has no toxicity or other side effects in animals (83). Thus, it can be used as a potential natural substitute for antibiotics.

### 2.5. *Epimedium* polysaccharide

**Figure 5:**
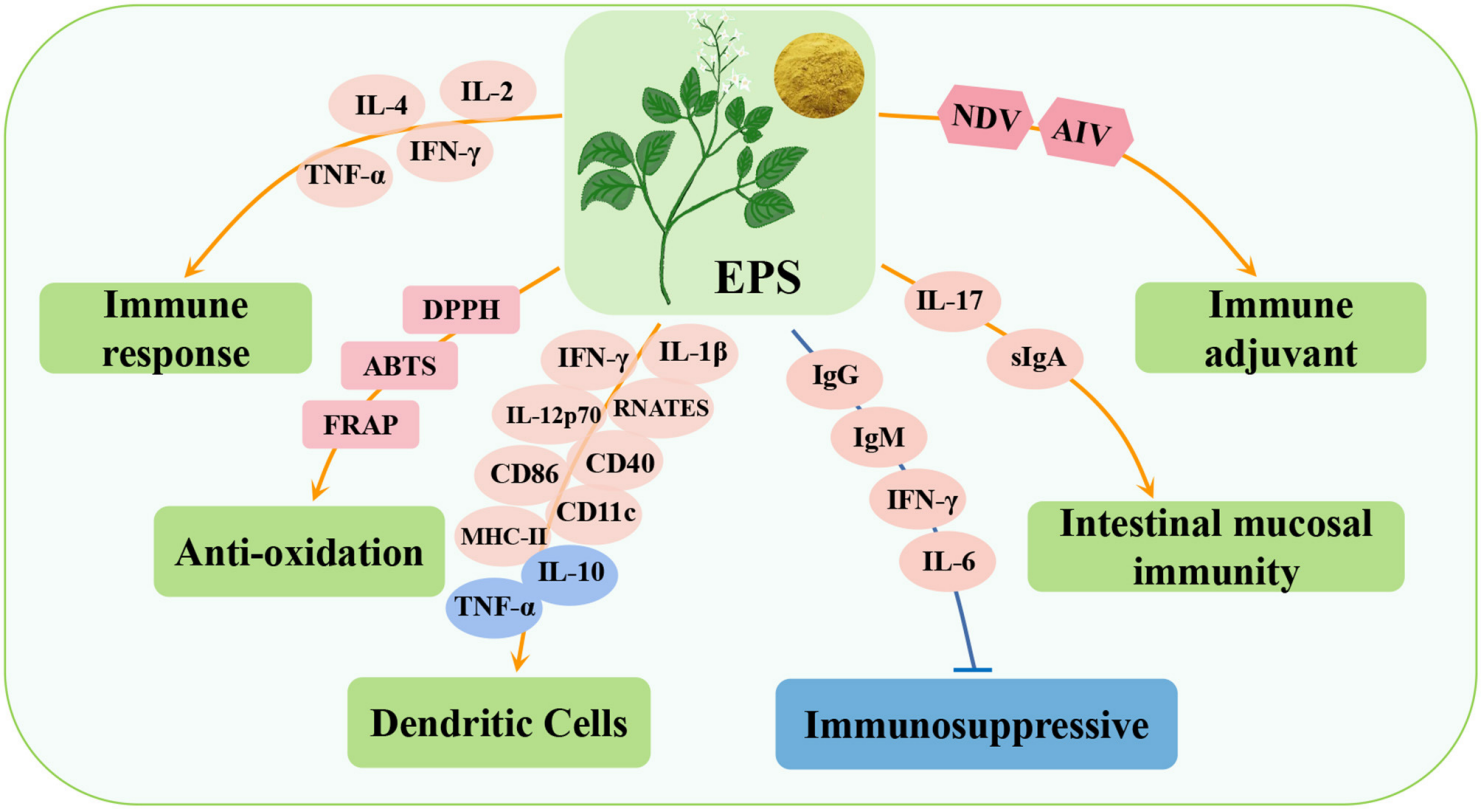
Immunomodulatory effects of *Epimedium* polysaccharide (EPS) in poultry. FRAP, ferric ion reducing antioxidant power; IL-12p70, interleukin-12p70; RNATES, regulated on activation normal T-cells expressed and secreted; CD86, CD86 molecule; CD40, CD40 molecule; CD11c, integrin subunit alpha X.

*Epimedium* polysaccharide (EPS) is one of the main active components of *Epimedium* and can be extracted by hot water extraction, alcohol precipitation and ethanol extraction (90, 91). It is mainly composed of mannose, rhamnose, glucuronic acid, galactosamine, glucose, galactose, arabinose, and fructose (92). EPS has many beneficial properties, such as antioxidation (93), anti-tumor (94), immunomodulation (95), antimicrobial (93), and anti-inflammation (96) (Table 5). Studies have shown that EPS has an immunomodulatory effect on poultry and can alleviate immunosuppression (Figure 5).

**Table 5:** Structure and structure-activity-relationship of polysaccharide from *Epimedium*.

**Type**	**Extraction method**	**Monosaccharide composition**	**Molecular weight/kDa**	**Structure characteristics**	**Function**	**References**
*Epimedium*	Hot water extraction	Rha: Glc: Gal: Ara=1.47: 6.50: 1.90: 0.76	21.90 kDa	4-Glc*p*, 3,6-Gal*p*, 3-Rha*p*, 3-Gal*p*, 4,6-Glc*p*, t-Ara*f*, t-Glc*p*	Anti-oxidation	(91)
*Epimedium*	Alcohol precipitation	GalA: Gal: Rha: Ara: GlcA=56.7%: 19.4%: 16.1%: 5.9%: 2.0%	-	-	Anti-oxidation	(92)
*Epimedium*	Hot water extraction	Glc: Gal: Man: Rha=4.68: 1.46: 3.60: 0.26	138.88 kDa	-	Anti-oxidation	(93)
*Epimedium*	Hot water extraction	Glc: Gal: Man: Xyl: Rha: Ara=4.36: 3.77: 0.29: 1.02: 0.34: 0.23	114.67 kDa	-	Antimicrobial	(93)
*Epimedium*	Hot water extraction	Man: Rha: GlcA: GalN: Glc: Gal: Ara: Fuc=1.00: 10.97: 0.43: 7.41: 2.77: 10.34: 11.33: 1.28	1800.00 kDa	28.20% uronic acid pyranoid ring	Anti-tumor	(94)
*Epimedium*	Ethanol extraction	GlcA: Gal: Rha=12.00: 1.00: 1.00	150.00 kDa	1,4-linked α-D-Gal*p*A, 1,3,4-linked α-D-Gal*p*A, 1,6-linked β-D-Gal*p* and terminal α-L-Rha*p* residues	Immunomodulation	(95)

Studies have shown that EPS can activate specific and non-specific immune responses in poultry. It promotes the proliferation of spleen lymphocytes and the secretion of immune cytokines (IL-2, IL-4, IFN-γ, and TNF-α), thus enhancing the specific immune response (95). Simultaneously, EPS can promote the maturation of the most powerful antigen-presenting dendritic cells in non-specific immunity, regulate the secretion of related factors (increasing the secretion of IIL-1β, IL-12p70, IFN-γ, and RNATES, and inhibiting the secretion of IL-10 and TNF-α), and promote cell maturation by increasing the expression of key surface molecules (MHC-II, CD40, and CD86) and inhibiting dendritic cell phagocytosis (97). In addition, EPS has strong scavenging ability for DPPH and ABTS free radicals and strong iron reduction ability in ferric ion reducing antioxidant power (FRAP) experiments, indicating that EPS has strong antioxidant ability and has the potential to be developed as a natural antioxidant (90, 91). Studies have also shown that EPS can enhance the immunity of intestinal mucosal cells by promoting IL-17 and sIgA secretion. IL-17 plays an important role in protection from both extracellular bacteria and fungi, as well as viruses that infect cells of the mucosal tracts. sIgA is the main immunoglobulin in the intestinal mucosa, which can kill harmful bacteria, neutralize the virus and prevent the virus from adsorbing to susceptible cells (97, 98). In addition, in the immunosuppressive model, the synergistic immune effect of the mixture of *Epimedium* polysaccharides and propolis flavonoids (EPA) was higher than that of propolis flavonoids alone, which could promote lymphocyte proliferation, up-regulate the titers of antibody, IFN-γ, IL-6, IgG and IgM, and promote the development of immune organs of CTX immunosuppressed chicks (99). Similarly, a large number of studies have shown that EPA can significantly inhibit Newcastle disease virus (NDV) and AIV infections in poultry cells and improve the antibody titer of the vaccine (100–102). These findings suggest that EPS has a significant effect on immune regulation in poultry.

### 2.6. *Salvia miltiorrhiza* polysaccharides

**Figure 6:**
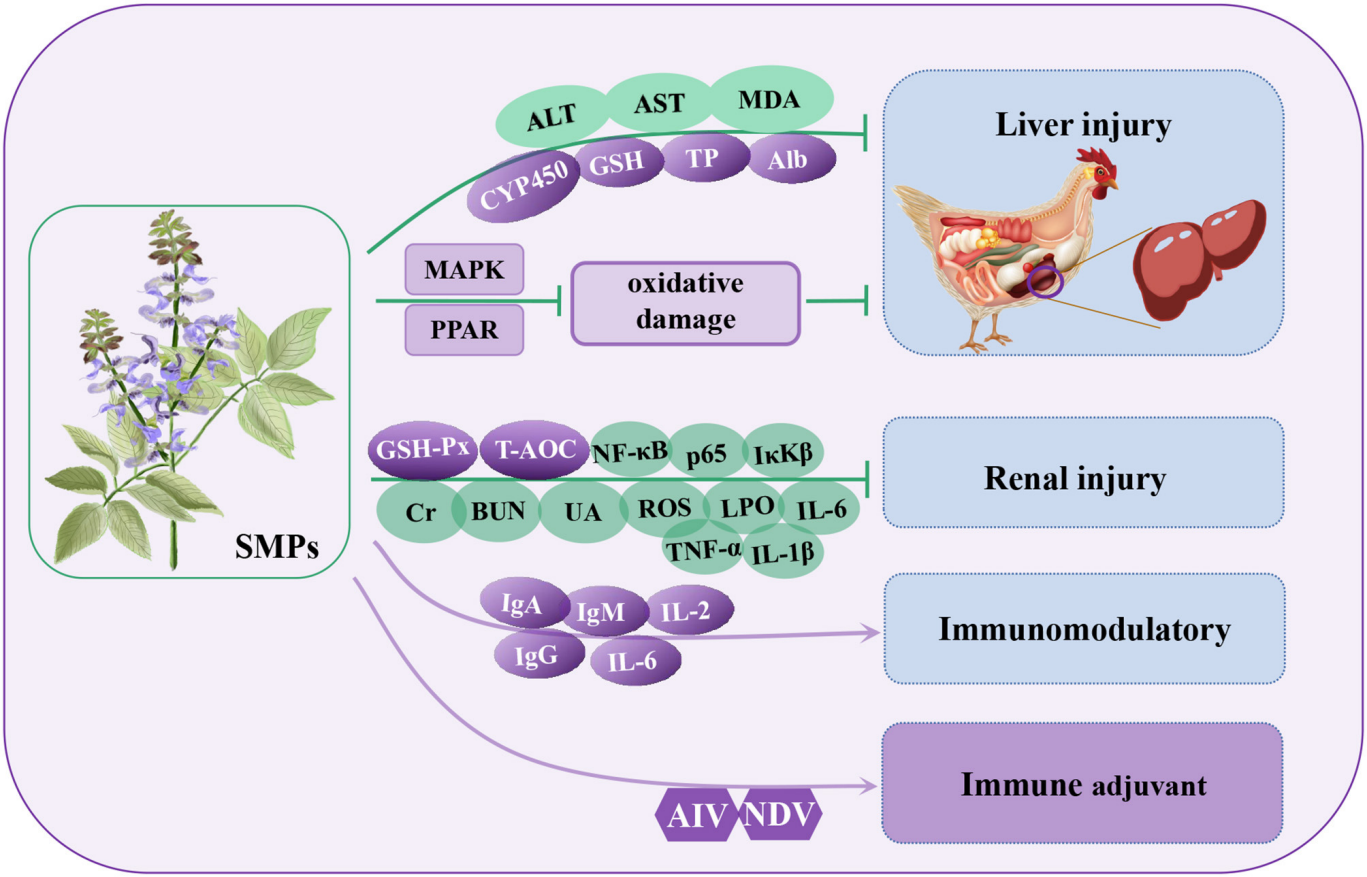
Regulatory mechanisms of *Salvia miltiorrhiza* polysaccharides (SMPs) in organ protection in poultry. ALT, glutamic pyruvic transaminase; AST, glutamic oxaloacetic transaminase; CYP450, cytochrome P450; TP, total protein; Alb, albumin; MAPK, mitogen activated kinase-like protein; PPAR, peroxisome proliferator activated receptor alpha; BUN, blood urea nitrogen; Cr, creatinine; UA, uric acid; LPO, lipid peroxide; IκKβ, inhibitor kappa B kinaseβ.

*Salvia miltiorrhiza* polysaccharides (SMPs) are among the main active components of the dried roots and rhizomes of *Salvia miltiorrhiza* and can be extracted by hot water extraction, ultrasound-assisted complex enzyme method, and microwave-assisted extraction (103–105). The components of the different extraction methods are slightly different and mainly composed of natural polymers, such as glucose, galactose, fructose, arabinose, mannose, and galacturonic acid (103, 105). SMPs are widely used to treat many diseases because of their various biological activities such as antivirus (106), intestinal immunity (105), anti-inflammation (107), anti-tumor (104), anti-oxidation (108), and liver protection (109) (Table 6). Studies have shown that SMPs can promote the proliferation of mouse T lymphocytes by activating the TLR-mediated MAPK and NF-κB signaling pathways and play an immunomodulatory role (110, 111). In addition, SMPs can improve rat mastitis induced by *Staphylococcus aureus* by inhibiting the activation of the NF-κB and MAPK signaling pathways (107). In recent years, an increasing number of researchers have studied the role of SMPs in the immune regulation of poultry and their potential mechanism (Figure 6).

**Table 6:** Structure and structure-activity-relationship of polysaccharide from *Salvia miltiorrhiza*.

**Type**	**Extraction method**	**Monosaccharide composition**	**Molecular weight/kDa**	**Structure characteristics**	**Function**	**References**
*Salvia miltiorrhiza*	Microwave assisted extraction	Glc: Gal: Fru=1.00: 1.67: 1.12	6.09 kDa	β-D-fru*f*, α-D-gal*p*, α-D-glc*p*	Relieve ferroptosis	(103)
*Salvia miltiorrhiza*	Hot water extraction	Gal: Glc: GalA=15.03: 7.14: 1.00	120.00 kDa	-	Anti-oxidation and anti-tumor	(104)
*Salvia miltiorrhiza*	Ultrasound-assisted complex enzyme method	Ara: Fru: Man: Glc: Gal=0.37: 0.41: 0.62: 3.21: 5.39	507.00 kDa	β-D-Fru*f*, β-D-Glc*p*, α-D-Gal*p*, β-L-Xyl*p*, α-D-man*p*, α-D-Glc*p*	Intestinal immunity and anti-oxidation	(105)
*Salvia miltiorrhiza*	Hot water extraction	Glc: Xyl: Man: Gal=0.34: 0.28: 0.27: 0.11	527.00 kDa	C-O-C and C-O-H	Anti-oxidation	(108)
*Salvia miltiorrhiza*	Hot water extraction	Ara: Gal: Glu: Rha: GalA=4.79: 8.24: 3.26: 1.00: 6.52	-	-	Immunomodulation	(110)

In a poultry kidney injury model, SMPs can alleviate inflammation by reducing the phosphorylation levels of NF-κB, p65, and IκKβ proteins and reducing the secretion of inflammatory factors (TNF-α, IL-1β, and IL-6) (112). Similarly, after feeding broilers with SMPs (0.50%), the content of immunoglobulin (IgG, IgM, and IgA) in serum increased, promoted the secretion of cytokines IL-2 and IL-6, and the antibody levels of ND and AI increased by 28.87 and 43.59%, respectively, thus improving the immune function of chicks (113). In a poultry hepatocyte injury model, SMPs can alleviate liver injury and metabolic disorders through drug metabolism induced by cytochrome P450 and the glycine, serine, and threonine metabolism signaling pathways (114). Through transcriptome and proteome studies, Geng et al. (115) found that SMPs alleviate oxidative stress and injury in broiler livers through the PPAR signaling pathway, MAPK signaling pathway, and glutathione metabolism (115). After poultry liver injury, the contents of total protein (TP), albumin (Alb), and GSH increased significantly, while after SMPs treatment, the contents of ALT, AST, and MDA decreased significantly, indicating that SMPs have a good protective effect on poultry liver injury *in vivo* and *in vitro* (115, 116). In addition, the strength of immunity is closely related to that of the kidneys. In a renal injury model, SMPs alleviated renal injury by regulating blood urea nitrogen (BUN), creatinine (Cr), uric acid (UA), upregulating antioxidant enzymes GSH-Px and serum T-AOC, rapidly eliminating lipid peroxide (LPO) and ROS, and reducing oxidative stress in the kidney (117). Other studies have shown that SMPs combined with florfenicol (FFC) can improve the growth performance of broilers, increase the number of leukocyte subtypes in blood, increase the number of immunoglobulins (IgG, IgM, and IgA) and cytokines (IFN-γ and IL-2) in serum, and stimulate T cell proliferation and differentiation to activate cellular immunity. It can also increase antibody titers against ND and AI (118). These findings suggest that SMPs have a significant ability to protect the liver and can be used as potential therapeutic drugs for the treatment of liver disease in poultry.

### 2.7. *Agaricus blazei* Murill polysaccharides

**Figure 7:**
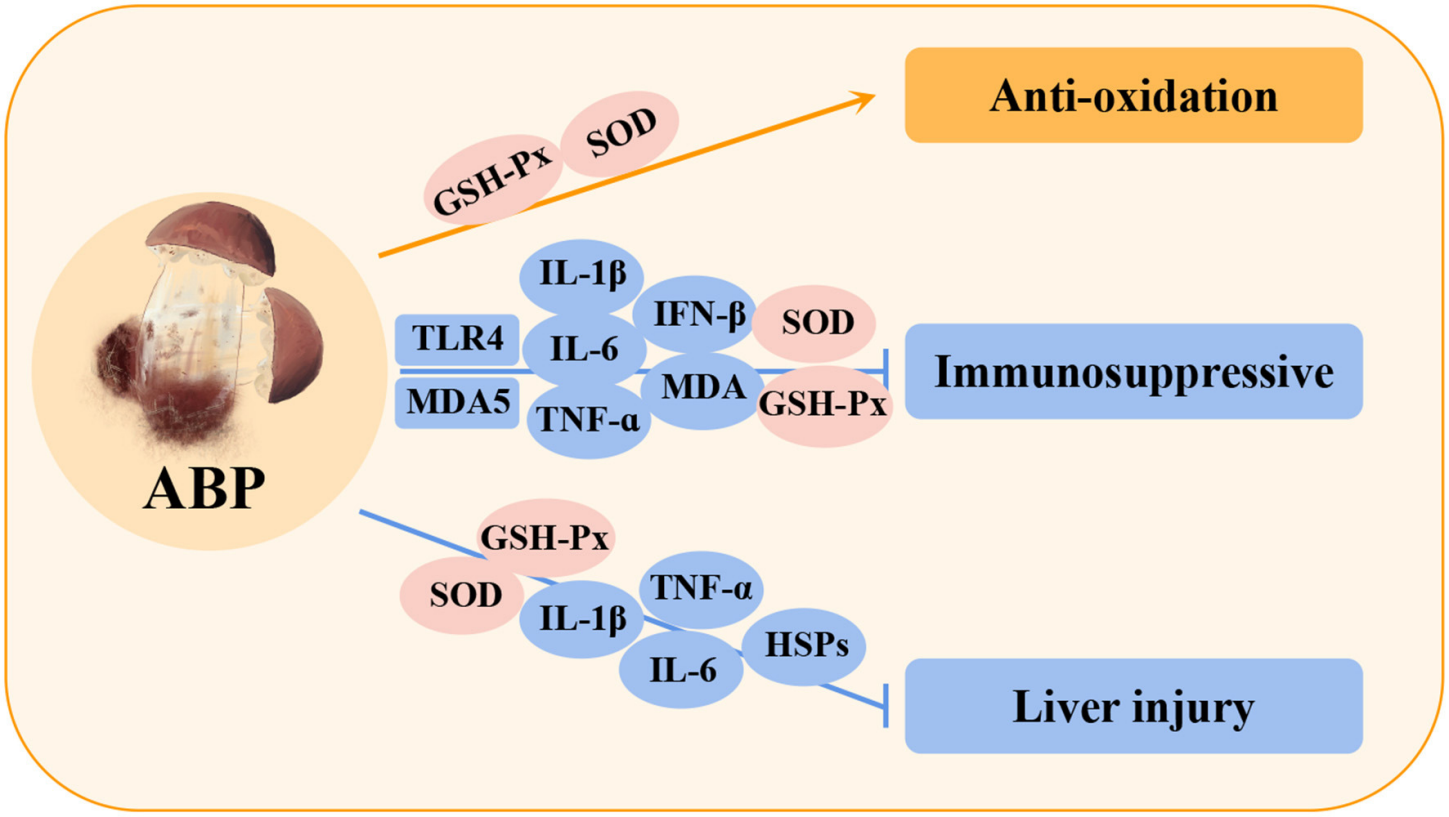
Immunomodulatory and hepatoprotective effects of *Agaricus blazei* Murill polysaccharides (ABPs) in poultry. C4, complement component 4; IFN-β, interferon-beta; HSPs, heat shock proteins.

*Agaricus blazei* Murill polysaccharides (ABPs) are among the main bioactive components of *Agaricus blazei* Murrill, and their functional properties have been recognized worldwide. These polysaccharides can be extracted by alcohol precipitation, microwave extraction, and hot water extraction (119–121). The types of polysaccharides produced by different extraction methods vary and mainly include glucose, mannose, galactose, rhamnose, arabinose (122, 123). Several experiments have shown that ABPs has many biological functions, such as anti-tumor (124), anti-inflammation (125), anti-aging (123), anti-oxidation (126), hypolipidemia (122), and anti-parasite (127) functions (Table 7). ABPs can significantly enhance immunity by activating and strengthening all types of immune cells, repairing abnormal cells, increasing the activity of various biological enzymes in cells, and improving the detoxification ability of the liver (128). Recent studies have shown that ABPs can play a positive role in the immune regulation of poultry *via* many pathways (Figure 7).

**Table 7:** Structure and structure-activity-relationship of polysaccharide from *Agaricus blazei* Murill.

**Type**	**Extraction method**	**Monosaccharide composition**	**Molecular weight/kDa**	**Structure characteristics**	**Function**	**References**
*Agaricus blazei* Murill	Alcohol precipitation	Glc were the only product	7340.00 kDa	α-(1 → 4)-D-glucopyranan with O-6 position occasionally occupied with α-Glc*p*-(1 → or α-Glc*p*-(1 → 6)-β-Glc*p*-(1 → sidechains	Immunomodulation	(119)
*Agaricus blazei* Murill	Microwave extraction	Glc: Man: Gal=9.92: 0.02: 0.06	1150.00 kDa	2,4,6-tri-O-Me-Glc; 2,3,4-tri-O-Me-Glc; 2,3,6-tri-O-Me-Gal; 2,3,4-tri-O-Me-Man; 2,3,4,6-tetra-O-Me-Glc	Immunomodulation	(120)
*Agaricus blazei* Murill	Hot water extraction	Glc: GlcA: Gal: Xyl: Fuc=9.51: 0.37: 0.09: 0.02: 0.01	10.00 kDa	β-1,6-D-Glc*p*, 1,3-GLC*p*, 1,4-Glc*p*, 1,3,6-Glc*p*, 1,2,4-Glc*p*, 1,3,4,6-Glc*p*, 1,6-Gal*p*	Hypolipidemia	(122)
*Agaricus blazei* Murill	Alcohol precipitation	Rha: Myo: Man: Glc=1.30: 1.10: 1.00: 2.00	144.00 kDa	-	Anti-oxidation	(123)
*Agaricus blazei* Murill	Alcohol precipitation	Glc were the only product	2000.00 kDa	l,4-glucopyranose and a small amount (1.5%) of non-starch α-glucan	Immunomodulation	(128)

Studies have shown that ABPs can significantly increase the contents of GSH-Px and SOD in poultry lymphocytes, indicating that ABPs helps to improve the antioxidant capacity and resistance of the body (129). In addition, in the immunosuppressive model of Cd, ABPs were shown to reduce the accumulation of Cd in chicken serum and immune organs and reduce Cd-induced damage by inhibiting MDA5 and TLR4 signaling pathways (130), which reduces the expression of downstream inflammatory factors (IL-1β, IL-6, TNF-α, and IFN-β), and ABP can promote the expression of anti-apoptosis protein (Bcl-2) and reduce the expression of caspase-3 and pro-apoptosis protein (Bax). It also decreased the levels of heat shock proteins (HSP60, HSP70, and HSP90) in spleen and increased the activity of antioxidant enzymes (SOD, and GSH-Px), indicating that ABP could improve the immunotoxicity induced by Cd (131, 132). In a model of liver injury, ABPs significantly reduced inflammatory cytokines (TNF-α, IL-1β, and IL-6) and HSPs (HSP27, HSP40, HSP60, HSP70, and HSP90) by increasing the activities of SOD and GSH-PX antioxidant enzymes, indicating that ABPs can effectively reduce oxidative stress and inflammation in liver injury by improving the antioxidant capacity (133). In summary, these studies demonstrated that ABPs exert their biological activity by regulating the immune system in poultry and mediating the protection of various organs (134).

### 2.8. *Enteromorpha* polysaccharide

**Figure 8:**
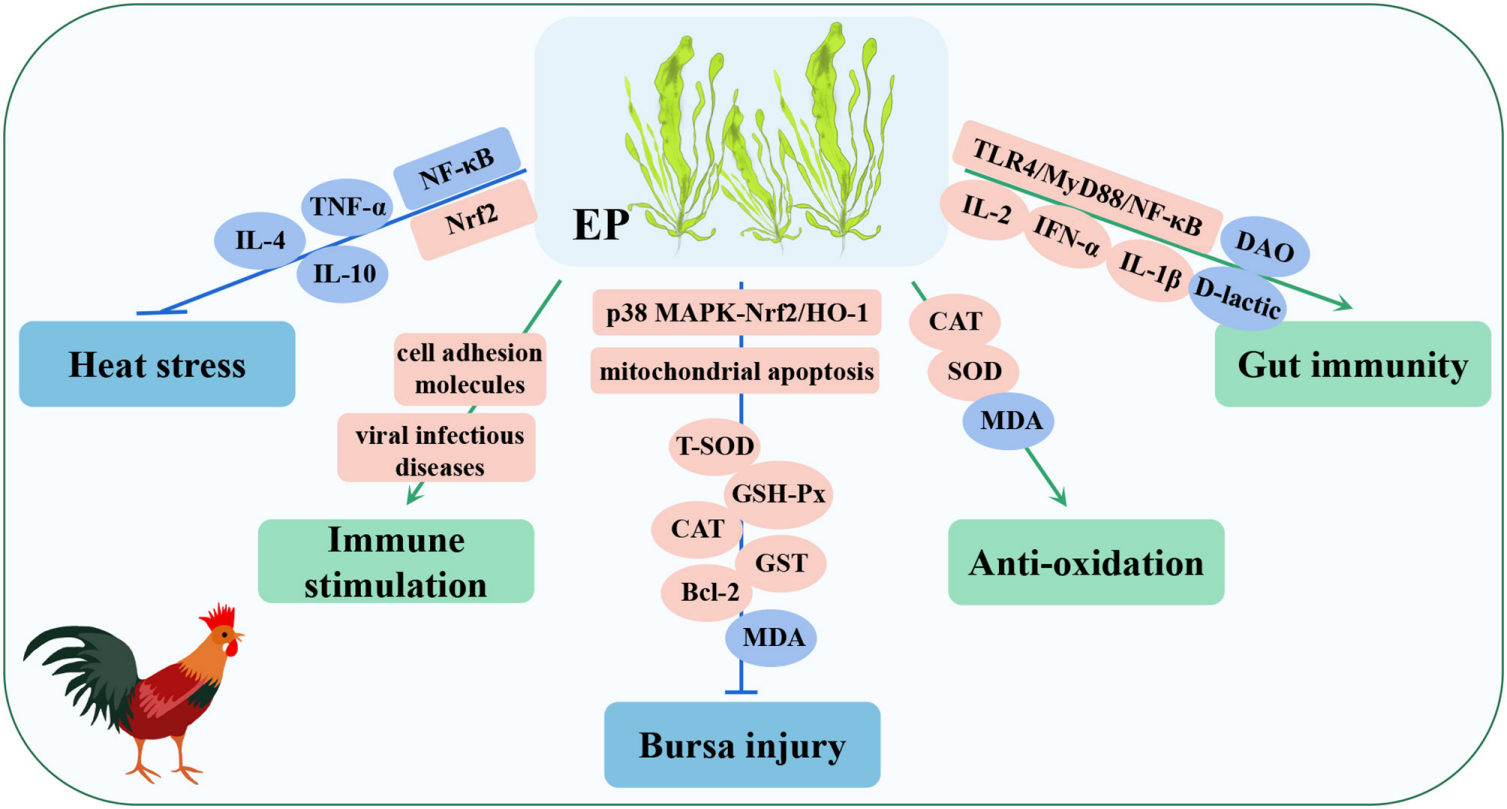
Immunomodulatory and disease-resistant effects of *Enteromorpha* polysaccharide (EP) in poultry. Nrf2, nuclea factor erythroid-2-related factor 2; Bcl-2, B-cell lymphoma-2; GST, glutathione-S-transferase; IFN-α, interferon-α.

*Enteromorpha* polysaccharide (EP) is one of the main active components of *Enteromorpha*, which is a natural wild green algae that is widely distributed in the East China Sea and South China Sea and represents a problematic genus along the coastal areas of China (135). In recent years, studies have found that *Enteromorpha* species have a variety of uses showed that EP has a variety of biological functions, including antiviral (136), anti-tumor (137), immune regulation (138), anti-oxidation (139), anti-diabetes (140), and blood lipid reduction (141). EP is mainly extracted by acid extraction (142), hot water extraction (143) and microwave-assisted extraction (139), and it is mainly composed of rhamnose, glucuronic acid, glucose, galactose, xylose, and mannose. EP structure is rich in sulfate (Table 8). In recent years, researchers have found that EP enhances immune regulation and disease resistance in poultry (Figure 8).

**Table 8:** Structure and structure-activity-relationship of polysaccharide from *Enteromorpha*.

**Type**	**Extraction method**	**Monosaccharide composition**	**Molecular weight/kDa**	**Structure characteristics**	**Function**	**References**
*Enteromorpha*	Hot water extraction	Rha: Glc: Xyl: Gal: GlcA=4.90: 1.80: 1.30: 0.90: 1.40	55.00 kDa	6.00% sulfate	Antiviral	(136)
*Enteromorpha*	Ethanol extraction	Rha: Xyl: Gal: Glc=5.36: 1.00: 0.57: 0.64	46.80 kDa	9.60% sulfate	Anti-tumor	(137)
*Enteromorpha*	Extraction with sodium hydroxide	Rha: Xyl: Man: Glc: Gal=2.40: 1.00: 0.23: 0.21: 0.18	-	17.40% sulfate	Immunomodulation and anti-oxidation	(138)
*Enteromorpha*	Microwaveassistance acid hydrolysis method	Rha: Man: Glc: Gal: Xyl: Fuc=1.00: 0.05: 1.03: 0.09: 0.28: 0.01	3.10 kDa	24.40% sulfate	Anti-oxidation	(139)
*Enteromorpha*	Enzymatic hydrolysis	Rha: GlcA: Glc: Xyl: Gal=4.16: 3.60: 2.07: 1.00: 0.46	44.10 kDa	-	Anti-diabetes	(140)
*Enteromorpha*	Water extraction-alcohol precipitation method	Rha: GlcA: Ara: Fuc: Xyl: Glc=5.12: 1.32: 3.38: 1.62: 1.00: 1.03	-	α-glucosidic bonds	Hypolipidemia	(141)
*Enteromorpha*	Acid extraction	Rha: GlcA: Glc: Xyl=1.00: 0.37: 1.16: 0.23	41.10 kDa	16.20% sulfate	High iron(III) chelating capacity	(142)
*Enteromorpha*	Hot water extraction	Rha: Glc: Gal: Xyl: Ara=1.48: 1.00: 0.13: 0.30: 0.06	147.80 kDa	15.53% sulfate	Anti-oxidation	(143)
*Enteromorpha*	Enzymatic hydrolysis	GlcN: Glc: GalA: Man: Xyl: Gal: Ara: GlcA: Fuc: GalN: Rib=5.08: 2.77: 1.17: 0.28: 0.26: 0.20: 0.09: 0.08: 0.03: 0.02: 0.02	1156.00 kDa	19.87% sulfate	Immunomodulation	(144)

The results showed that the addition of EP to the diet of poultry increased the activity of SOD in the liver and CAT in the serum, decreased the level of MDA in the serum and liver, and enhanced the ability of antioxidation (145, 146). Similarly, after including EP in the diet of poultry, the pectoral muscles and bursa of Fabricius were sampled for transcriptome sequencing (RNA-seq), and a GO analysis showed that the differentially expressed genes (DEGs) were mainly enriched in immune-related signaling pathways, while a KEGG analysis indicated that they were enriched in viral infectious disease, cell adhesion molecule, and other immune signaling pathways, and these findings indicated that EP mainly plays an immunostimulatory role in poultry (147, 148). Moreover, EP can increase the mRNA expression of IL-2, IFN-α, and IL-1β in intestinal tissue by activating the TLR4/MyD88/NF-κB signaling pathway, and significantly decrease the level of D-lactic acid and the activity of DAO in serum, both of which reduce intestinal barrier functions and permeability. Simultaneously, EP also increased the abundance of *Bacteroides* and *Lactobacillus*. The short-chain volatile fatty acids (SCFAs) produced by these bacteria are involved in the immune response and intestinal barrier function of the body, indicating that EP can improve the intestinal immune response and integrity of poultry (149, 150). In a heat stress model, EP alleviated the imbalance of Th1/Th2 cells by activating the Nrf2 signaling pathway, inhibiting the NF-κB signaling pathway, and reducing the expression of proinflammatory cytokines (TNF-α, IL-4, and IL-10), thus improving the inflammatory response and injury of immune organs induced by heat stress (144, 151). In addition, in a poultry immune organ bursa oxidative injury model, EP reduced bursa cell necrosis by activating the p38 MAPK-Nrf2/HO-1 and mitochondrial apoptosis signaling pathways, increased the activities of antioxidant enzymes (T-SOD, GSH-Px, CAT, and GST), upregulated the expression of antioxidant and apoptosis-related proteins (Nrf2, HO-1, p38 MAPK, Bcl-2), and decreased the content of MDA. As a result, the antioxidant capacity of the bursa of Fabricius was enhanced and injury was reduced (152). These studies showed that EP has strong immunomodulatory and antioxidant capacities and can be used as a nutritional supplement as an antioxidant and immune enhancer, thereby providing theoretical support for the development and utilization of *Enteromorpha* resources.

### 2.9. *Glycyrrhiza* polysaccharides

**Figure 9:**
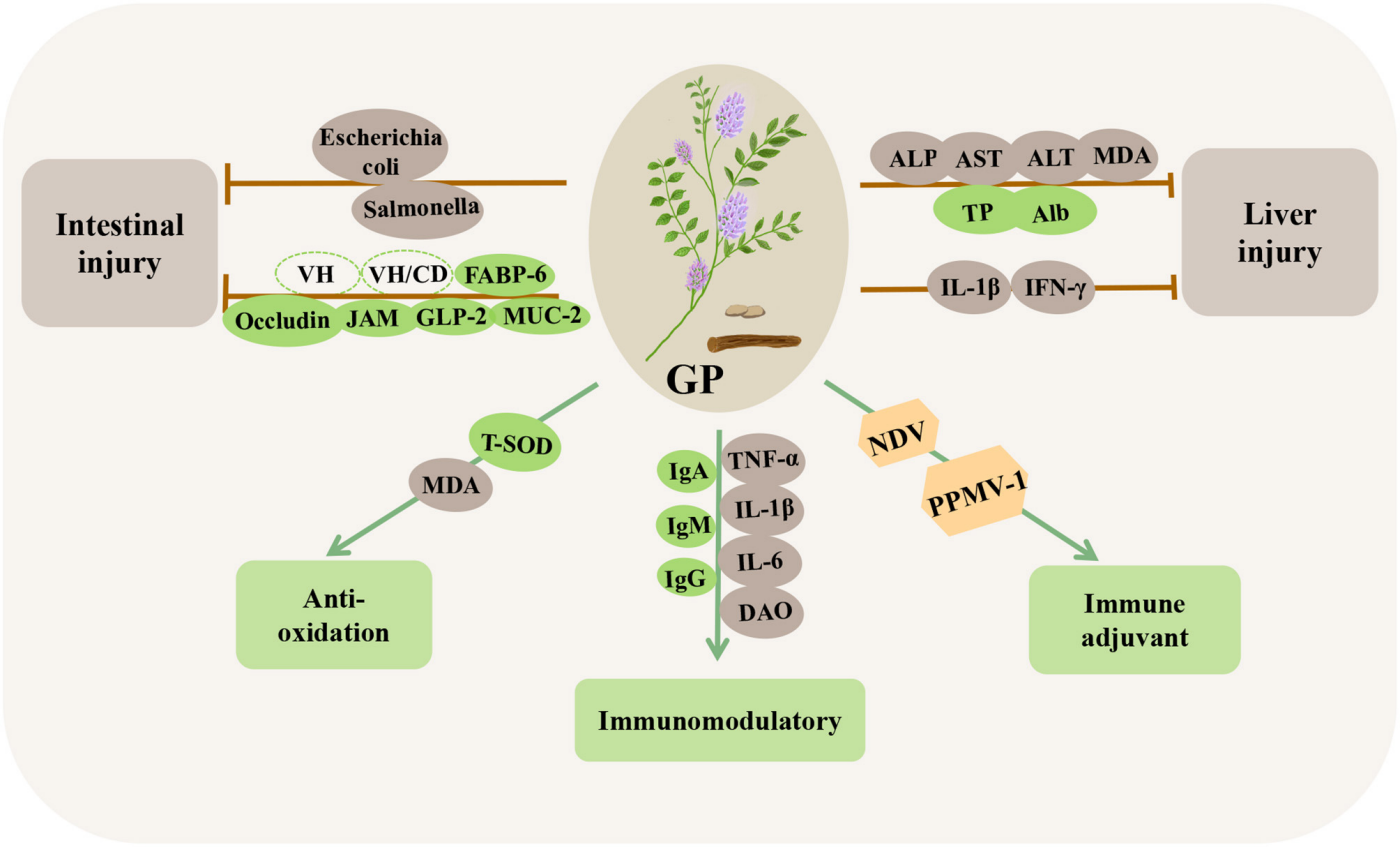
Immunomodulatory and disease-resistant effects of *Glycyrrhiza* polysaccharides (GPs) in poultry. VH, villus height; VH/CD, villus height/crypt depth; FABP-6, fatty acid binding protein 6; Occludin, tight junction proteins; JAM, junction adhesion molecules; GLP-2, glucagon-like peptide-2; MUC-2, mucin-2; PPMV-1, paramyxovirus type 1; ALP, alkaline phosphatase.

*Glycyrrhiza* polysaccharides (GPs) are among the main bioactive components of licorice, and they are mainly composed of arabinose, glucose, galactose, rhamnose, mannose, xylose, and galacturonic acid in different proportions and with different types of glycosidic bonds (153–155). GPs is considered a potential immune enhancer for poultry because of its biological activities, such as antioxidation (156, 157), immune regulation (158, 159), anti-tumor (160), and intestinal flora regulation (161) (Table 9; Figure 9).

**Table 9:** Structure and structure-activity-relationship of polysaccharide from *Glycyrrhiza*.

**Type**	**Extraction method**	**Monosaccharide composition**	**Molecular weight/kDa**	**Structure characteristics**	**Function**	**References**
*Glycyrrhiza*	Hot water extraction	Glc: Gal: Ara=2.34: 2.52: 0.83	10.16 kDa	-	Anti-oxidation	(153)
*Glycyrrhiza*	Alkali extraction	Rha: Ara: Xyl: Man: Glc: Gal=1.00: 2.33: 2.85: 0.69: 3.05: 1.54	2890.00 kDa	→ 6)-β-D-Glc*p*-( → backbone and the → 4)-α-D-Xyl*p*-(1 → , → 5)-α-L-Ara*f*-(1 → , → 3)-α-L-Rha*p*-(1 → , → 6)-α-D-Gal*p*-(1 → , → 3,6)-α-Man*p*-(1 → and → 1)-β-D-Glc*p* as branches	-	(154)
*Glycyrrhiza*	Hot water extraction	Xyl: Man: Glc: Gal=0.22: 1.20: 0.22: 1.00	-	-	Anti-oxidation	(155)
*Glycyrrhiza*	Hot water extraction	Ara: Man: Glc: Gal=1.00: 0.49: 4.05: 3.17	1960.00 kDa	Main chain α-D-1,4-Glc, α-D-Glc-1,3,6 and α-D-Glc-1,2,3,6-Glc, side chain α-D-1,3 and β-L-1,2-Ara, Gal, Man	Anti-oxidation	(156)
*Glycyrrhiza*	Hot water extraction	Glc: Ara: Man: Gal=9.80: 0.05: 0.07: 0.07	38.70 kDa	1,4-linked α-D-Glc*p*, 1,6-linked α-D-Glc*p*, 1,4-linked to main side α-D-Glc*p* and terminal 1-linked β-D-Glc*p* to the C-3 of residue D	Anti-oxidation	(157)
*Glycyrrhiza*	Hot water extraction	Rha: Ara: Man: Glc: Gal=1.00: 13.87: 1.59: 16.76: 15.72	29.10 kDa	α-L-Ara*f*-(1-,3)-α-L-Rha-(1-,3)-α-D-Gal*p*-(1-,α-D-Xyl*p*-(1-, and-4)-α-D-Glc*p*-(1-residue)	Immunomodulation	(158)
*Glycyrrhiza*	Hot water extraction	GalA: Ara: Rha: Gal: ManA: Glc: Fuc: Xyl=7.44: 1.01: 0.64: 0.48: 0.15: 0.13: 0.09: 0.05	26.40 kDa	→ 4)-α-D-Galp*A*-(1 → , → 2,4)-α-D-Galp*A*-(1 → , α-L-Ara*f*-(1 → , → 5)-α-L-Ara*f*-(1 → , → 4)-α-D-Gal*p*A, → 4)-β-D-Gal*p*A	Immunomodulation	(159)

Studies have shown that GPs can promote the development of immune organs and stimulate the continuous proliferation of spleen macrophages, significantly increase the levels of IgA, IgM, and IgG in the serum, and significantly reduce the levels of TNF-α, IL-1β, IL-6, and DAO to induce an immune response, thereby improving the immune function of the body (162–164). In a study on the antioxidation capacity of GPs, the direct addition of 1000 or 1500 mg/kg GPs to chicken diets significantly increased the T-SOD activity and decreased the serum MDA content (165). Similarly, the antioxidant effect of GPs plays a significant role in reversing oxidative stress induced by LPS and can reduce the increase in IL-1β and IFN-γ in the liver induced by LPS (166). In addition, GPs significantly alleviated oxidative stress in broilers induced by mycotoxins, significantly decreased the activities of ALP, AST, and ALT and the concentration of MDA, and increased the concentration of serum total protein and albumin, thus alleviating liver injury (167). In intestinal immunity, dietary GPs can significantly increase the villus height and the ratio of villus height to crypt depth (163, 165) and significantly upregulate the mRNA expression of tight junction proteins (occludin), junction adhesion molecules (JAM), glucagon-like peptides-2 (GLP-2), fatty acid binding protein 6 (FABP-6), and mucin-2 (MUC-2) in the intestine (142, 164). Similarly, GPs can significantly reduce the number of *Escherichia coli* and *Salmonella* in poultry intestines, thereby enhancing intestinal immunity and maintaining healthy intestinal microorganisms (161). In addition, GPs can be used as a vaccine adjuvant, which can significantly increase chicken lymphocytes and reduce the copy number of viral RNA, thus enhancing the titer of NDV and paramyxovirus type 1 (PPMV-1) vaccines (168–170). These results suggested that GPs have immunomodulatory activity and can be used as potential innate immunomodulators.

### 2.10. Others

In addition to the above plant polysaccharides, other polysaccharides also have important immune regulatory activities in poultry (Table 10). Recent studies have shown that the addition of *Lycium barbarum* polysaccharide (LBP) to poultry diets can increase the enzyme activity of SOD and GSH-Px, reduce the content of MDA, increase the content of IgG and IgA in serum, and increase the cytokine levels of TNF-α, IL-4, IL-6, and IFN-γ to improve the antioxidation ability and enhance immune function (171). Similarly, *Acanthopanax senticosus* polysaccharide (ASPS) can improve the activities of SOD and GSH-Px in serum, decrease the content of MDA, and significantly increase the content of IgA and IgM in serum, thereby improving the antioxidant capacity and immune status (172). In addition, ASPS can promote lymphocyte proliferation, increase the CD4^+^ and CD8^+^T lymphocyte proportion, and promote IFN-γ and IL-2 secretion, indicating that ASPS can enhance immune activity in poultry (173). In addition, Yingshan yunwu tea polysaccharide (YYTP) could increase the immune organ index and the secretion of immunoglobulin (IgA and IgG) in poultry. YYTP has strong DPPH radical, superoxide anion, and hydroxyl scavenging activities and enhances the T-AOC, SOD, and GSH-Px activity. These results indicate that YYTP has strong antioxidant capacity, can be used as a natural antioxidant, and has a positive role in promoting immune response (174, 175). A previous study reported that ginseng polysaccharide (GPS) stimulates immune cells to secrete cytokines (IL-2, IL-10, IFN-β, and TNF-α) and increases antibody titers of H5N1 avian influenza virus vaccine, thus demonstrating its potential in improving the immune response (176). Similarly, Mulberry leaf polysaccharide (MLP) can significantly stimulate lymphocyte proliferation and promote the secretion of IL-2, IFN-γ, sIgA, IgG, and IgA, thereby improving the antibody titer of ND in poultry serum. Moreover, it can be used as a potential immune adjuvant for ND (177, 178).

**Table 10:** Plant polysaccharide biological activity and mechanisms.

**Plant polysaccharides**	**Biological activities**	**Mechanisms involved**	**References**
*Lycium barbarum* polysaccharide (LBP)	Antioxidative activity/Immunomodulatory activity	Increase activity of SOD, GSH-PX. Decrease in MDA. Increase in TNF-α, IL-4, IL-6 and IFN-γ.	(171)
*Acanthopanax senticosus* polysaccharide (ASPS)	Antioxidative activity/Immunomodulatory Activity/Resist immunosuppression	Increase activity of SOD, GSH-PX. Decrease in MDA. Increase lymphocyte proliferation and cytokine (IFN-γ and IL-2) secretion.	(172, 173)
Yingshan yunwu tea polysaccharide (YYTP)	Antioxidative activity/Immunomodulatory activity	Scavenge DPPH, superoxide anion and hydroxyl radicals. Increase activity of SOD, GSH-Px, T-AOC. Decrease in MDA. Increase immune organ index and immunoglobulin (IgA and IgG).	(174, 175)
*Ginseng* polysaccharide (GPS)	Immunomodulatory Activity/Immune adjuvant	Increase in IL-2, IL-10, IFN-β and TNF-α. Increase antibody titers of H5N1 vaccine.	(176)
Mulberry leaf polysaccharide (MLP)	Immunomodulatory Activity/immune adjuvant	Increase activity of IL-2, IFN-γ, sIgA, IgG, IgA. Increase the antibody titer of ND.	(177, 178)
*Artemisia ordosica* polysaccharide (AOP)	Antioxidative activity/Anti-inflammation activity	Modulation Nrf2/Keap1 and TLR4/NF-κB signaling pathway. Increase activity of T-AOC, SOD, CAT, GPx. Decreases IL-1β, IL-6, TNF-α.	(179)
*Polygonatum sibiricum* polysaccharide (PP)	Resist immunosuppression	Regulates IL-2, IL-6 and IFN-γ. Increase IgG, IgM.	(180)
Bush sophora root polysaccharide (BSRPS)	Immunomodulatory Activity	Increase SOD2. Decrease CYP450 1A5.	(181)
*Echinacea purpurea* polysaccharide (EPP)	Immunomodulatory Activity	Increase IL-2, IFN-γ and CD80. Activation of dendritic cells.	(182)
*Platycodon grandiflorum* polysaccharide (PGPS)	Immunomodulatory Activity	Activates macrophages and T lymphocytes. Increase the expression of CD80, CD86, TNF-α, IL-1β, IL-6 in macrophages.	(183, 184)
*Paulownia fortunei* flower polysaccharide (PFFPS)	Immunomodulatory Activity	Increase lymphocytes and leukocyte. Increase IL-2, IFN-γ and sIgA.	(185)

In addition, *Artemisia ordosica* polysaccharide (AOP) can alleviate LPS-induced oxidative stress injury in poultry by activating the Nrf2/Keap1 signaling pathway, inhibiting the TLR4/NF-κB pathway, increasing the activity of antioxidant enzymes (T-AOC, SOD, CAT, and GPX), and reducing the expression of proinflammatory cytokines (IL-1β, IL-6, and TNF-α), thus indicating that AOP has antioxidant and anti-inflammatory effects (179). Shu et al. found that *Polygonatum sibiricum* polysaccharide (PP) can effectively alleviate immunosuppression injury by increasing the antibody content of IgG and IgM, stimulating the proliferation of T lymphocytes in peripheral blood, and regulating the expression levels of IL-2, IL-6, and IFN-γ (180). In addition, Gan et al. found that bush sophora root polysaccharide (BSRPS) could alleviate hepatocyte injury by improving the expression of SOD2 and downregulating CYP450 1A5 (181). In immune cells, *Echinacea purpurea* polysaccharide (EPP) can increase the expression of CD80 surface markers in poultry dendritic cells, increase the levels of IL-2 and IFN-γ, and stimulate immune cell proliferation, indicating that EPP can drive dendritic cells to mature and play an immunomodulatory role (182). Similarly, *Platycodon grandiflorum* polysaccharide (PGPS) can increase the proliferation of T lymphocytes and macrophages, increase the phagocytosis rate of macrophages and the expression of maturation markers (CD80 and CD86), and stimulate macrophages to produce the cytokines TNF-α, IL-1β, and IL-6, indicating that PGPS has significant immune activity (183, 184). In addition, *Paulownia fortunei* flower polysaccharide (PFFPS) can significantly increase leukocytes and lymphocytes, increase the contents of IL-2, IFN, and SIgA in the duodenum, and increase the antibody titer of ND (185). Taken together, plant polysaccharides have many beneficial effects in poultry immune regulation, such as enhancing immunity, anti-oxidation, anti-inflammation, resisting immunosuppression, and improving vaccine titer, but their potential regulatory pathways are still not completely clear.

## 3. Conclusions

The above data indicate that the molecular weight of PAMK is smaller than other polysaccharides, but similar to APS, it has more bioactive functions. TPPPS is widely studied in poultry antivirus and is a potential vaccine adjuvant. AP and SMPs have good protective effects on liver injury in poultry. Glucose plays an important role in immune function of ABPs. EP are different from the polysaccharides of terrestrial plants, their structure is rich in sulfate, and their biological functions are mainly sulfated polysaccharides. In general, plant polysaccharides play many important roles in poultry immune regulation, such as regulating the body's immune function, improving the antioxidant capacity, relieving immunosuppression, promoting the recovery and regeneration of immune organs, improving the regulation of cytokines, improving intestinal flora, and acting as potential vaccine adjuvants. Hence, plant polysaccharides represent a good and safe method of improving the immune regulatory function of poultry; moreover, plant polysaccharides as green additives may be used as potential therapeutic candidates to treat poultry-related diseases. However, certain concerns remain to be addressed:(1) Different plant polysaccharides have different origins, and the efficiency of immune regulation by polysaccharides from different origins must be compared and investigated. (2) The biological activity mechanism, dosage, effect, and higher-order structure of plant polysaccharides are unknown, which limits their development and utilization. (3) A single polysaccharide should be strengthened and improved for different purposes and combined in different proportions into a complex polysaccharide with multiple functions to improve the utilization rate and functional development of plant polysaccharides. (4) The immune regulatory mechanism of plant polysaccharides involves different receptors on various immune cells and has a regulatory effect on intercellular messenger molecules. Therefore, its mechanism of action must be explored at multiple levels. (5) The bioactive function and precise targeting of different modified plant polysaccharides and drug carrier systems (such as liposomes and nanoparticle carriers) should be improved to improve the therapeutic effect and reduce the dosage and side effects. Such improvements represent an important direction for future research on plant polysaccharides. (6) The potential application of plant polysaccharides in treating poultry diseases needs to be further explored. The current findings and future studies could provide more information on the regulatory and molecular mechanisms of plant polysaccharides in the development, regulation, and function of immune-related diseases of the poultry immune system.

## Author contributions

R-HZ: conceptualization, project administration, visualization, and writing—original draft. F-XY: project administration, visualization, and writing—original draft. Y-CB: project administration and visualization. J-YZ, MH, and X-YZ: project administration. T-FD: project administration and supervision. J-JJ: conceptualization, funding acquisition, supervision, and writing—review and editing. All authors contributed to the article and approved the submitted version.
